# Development of Fluorescence Polarization Immunoassay With scFv to Detect Fumonisin B_s_ in Maize and Simultaneous Study of Their Molecular Recognition Mechanism

**DOI:** 10.3389/fchem.2022.829038

**Published:** 2022-02-21

**Authors:** Yuan Li, Qing Yu, Wenbo Yu, Suxia Zhang, Kai Wen, Jianzhong Shen, Zhanhui Wang, Xuezhi Yu

**Affiliations:** College of Veterinary Medicine, China Agricultural University, Beijing Key Laboratory of Detection Technology for Animal-Derived Food Safety, Beijing Laboratory for Food Quality and Safety, Beijing, China

**Keywords:** fluorescence polarization immunoassay, scFv, fumonisin B_s_, maize, molecular recognition mechanism

## Abstract

In this study, a fluorescence polarization immunoassay (FPIA) was developed based on the single-chain variable fragments (scFvs) for fumonisin B_s_ (FB_s_). The scFvs were prepared from FB_s_-specific monoclonal antibody secreting hybridomas (4F5 and 4B9). The established FPIA could determine the sum of fumonisin B_1_ (FB_1_) and fumonisin B_2_ (FB_2_) within a short time. The IC_50_ of FPIA for the detection of FB_1_ and FB_2_ were 29.36 ng/ml and 1,477.82 ng/ml with 4F5 scFv, and 125.16 ng/ml and 30.44 ng/ml with 4B9 scFv, so the 4B9 scFv was selected for detection of FB_1_ and FB_2_ in maize samples with a limit of detection of 441.54 μg/kg and 344.933 μg/kg. The recoveries ranged from 84.7 to 104.1% with a coefficient of variation less than 14.1% in spiked samples, and the result of the FPIA method was in good consistency with that of HPLC-MS/MS. To supply a better understanding of the immunoassay results, the interactions mechanism of scFvs-FB_s_ was further revealed by the homology modelling, molecular docking, and molecular dynamic simulation. It was indicated that six complementarity-determining regions (CDRs) were involved in 4B9 scFv recognition, forming a narrow binding cavity, and FB_1_/FB_2_ could be inserted into this binding cavity stably through strong hydrogen bonds and other interactions. While in 4F5 scFv, only the FB_1_ stably inserted in the binding pocket formed by four CDRs through strong hydrogen bonds, and FB_2_ did not fit the binding cavity due to the lack of hydroxyl at C10, which is the key recognition site of 4F5 scFv. Also, the binding energy of FB_2_-4B9 scFv complex is higher than the FB_2_-4F5 scFv complex. This study established a FPIA method with scFv for the detection of FB_1_ and FB_1_ in maize, and systematically predicted recognition mechanism of FB_s_ and scFvs, which provided a reference for the better understanding of the immunoassay mechanism.

## Introduction

Fumonisins (FB_s_) produced by *Fusarium* pathogens, are mycotoxins present in maize and other grains during storage and pose a serious threat to humans and domestic animals worldwide. FB_s_ consist of different chemotypes, and fumonisin B_1_ (FB_1_) and fumonisin B_2_ (FB_2_) are believed to be the most prevalent and toxic in naturally contaminated cereals (Weidenbörner, 2001). Numerous guidance or regulations for FB_s_ have been enforced to protect public health. The Food and Drug Administration stated the maximum levels of total FB_1_, FB_2_, and fumonisin B_3_ (FB_3_) were 2–4 mg/kg in human food, and the European Union has established a maximum residue limit in human maize of 1 mg/kg FB_1_ + FB_2_ (Weidenbörner, 2001).

Many methods have been developed for FB_s_ detection, including the golden method of mass spectrometry (LC-MS) and tandem mass spectrometry (LC-MS/MS) for the simultaneous multiple mycotoxin analysis (Songsermsakul and Razzazi-Fazeli, 2008). Immunoassays, due to the advantage of high specificity, high efficiency, and low cost, become more and more popular in residue detection. Enzyme-linked immunosorbent assay (ELISA) as the most used immunoassay is a heterogeneous method in solid phase that is limited by incubation and washing steps (Sheng et al., 2012). Thus, much effort needs to be focused on some one-step homogeneous assay without multiple washing steps.

Fluorescence polarization immunoassay (FPIA) is a homogeneous assay and has been applied to detect numerous mycotoxins, including aflatoxins (AF_s_), FB_s_, deoxynivalenol (DON), T-2 toxin, ochratoxin A (OTA), and zearalenone (ZEA) (Zhang et al., 2017). Li et al. revealed a limit of detection (LOD) of 157.4 μg/kg for FB_1_ and 290.6 μg/kg for FB_2_ in the optimized FPIA with the pair of FB_1_-FITC and 4B9 traditional monoclonal antibody (mAb) (Li et al., 2015). However, these studies are based on mAbs whose production always takes several months. With the development of recombinant antibody expression technology, an increasing number of studies have been developed based on the recombinant antibodies due to their simple preparation process and without sacrifice of animals.

Single-chain variable fragments (scFvs), as one of the most conventional recombinant antibodies, contain the antibody variable regions of the light chain (VH) and variable regions of heavy chain (VL) and are always linked by a small, flexible peptide chain. ScFv can be generated by many techniques including ribosome display technology (Mahalakshmi et al., 2019), phage display technology (Hu et al., 2016), and also directly cloned from the obtained hybridoma cells. Cloning VH and VL from hybridoma cell lines is indicated to be a more directable and efficient method for scFv production (Liu et al., 2016).

The traditional method of the antibody-antigen interaction mechanism study is always based on a complete antibody three-dimensional (3D) structure analyzed by precise X-ray crystallography and nuclear magnetic resonance (NMR) techniques (Arata, 1991). However, these methods are complicated, time-consuming, and also limited by not being able to capture the dynamics of antibody (Srivastava et al., 2018). In recent years, computational methods proved to be immensely successful in understanding antibody dynamics in solution with the characteristic of economy and time-saving. Homology modelling has been widely used for 3D model construction of different proteins (scFv, enzyme, and receptor) (Liu et al., 2010), and molecular docking and molecular dynamics (MD) simulation were used to analyze the molecular interactions of these proteins with their respective ligands, such as haptens and ligands (Nencetti et al., 2011). However, the computational method could not accurately describe the structure information of real antibody structure as X-ray crystallography. The predicted structures also have become a promising method to underline protein dynamics in solution.

In this study, the anti-FB_s_ scFvs 4F5 and 4B9 were produced from related hybridoma cell lines. Then the FPIA was developed for the detection of FB_1_ and FB_2_ with 4B9 scFv in maize. Furthermore, to explain the different affinities of 4B9/4B5 scFv with FB_s_, homology modelling, molecular docking, and MD simulation were performed to study the interactions of scFvs-FB_1_ and scFvs-FB_2_. These data predicted the different recognition mechanisms between scFvs and FB_s_, which provided a better understanding of immunoassay results and guide further molecular evolution to generate affinity-improved antibodies.

## Materials and Methods

### Apparatus and Buffers

A Spectramax M5 microplate reader was supplied by Molecular Devices, LLC (Sunnyvale, CA, USA). Precoated TLC silica gel 60 aluminum sheets (F254) for thin-layer chromatography (plate size:10 × 10 cm; layer thickness: 0.15–0.2 mm, particle size: 2 μm) were acquired from Merck (Darmstadt, Germany). Black, opaque 96-well microplates with non-binding surfaces were supplied by Corning, Inc. (Oneonta, NY, USA). Syringe filters (0.45 μm) were obtained from Tianjin Jinteng Experiment Equipment Co., Ltd. (Tianjin, China).

Borate buffer (BB, pH 7.0) was used as a working diluent buffer and was prepared by mixing 200 mM boric acid (pH 5.38) and 50 mM sodium borate (pH 9.49) at a ratio of 47:3 (v/v). Lysis buffer (50 mM Tris, 300 mM NaCl, 1 mM EDTA, 0.5 mM PMSF, and 200 µg lysozyme, pH 8.0), washing buffer (50 mM Tris-HCl, 500 mM NaCl, and 25 mM imidazole, pH 8.0), and elution buffer (50 mM Tris-HCl, 150 mM NaCl, and 300 mM imidazole, pH 8.0) were used to purify scFv. 2 × YT medium was used to cultivate *Escherichia coli* and was prepared by mixing 1.6% tryptone, 1% yeast extract, and 0.5% sodium chloride.

The soluble protein expression vector pJB33 and the RV308 strain used in this study were obtained as a gift from the laboratory of Andreas Plückthun (Biochemical Institute, University of Zurich, Switzerland). The oligonucleotide primers were synthesized at GENEWIZ (Suzhou, China). The hybridoma cell lines 4F5 and 4B9 were previously established in our laboratory (Li et al., 2015).

### Reagents and Chemicals

The RNeasy Mini Kit and Ni NTA Agarose were obtained from QIAGEN, Inc. (Dusseldorf, Germany). The PrimeScript RT-PCR Kit and Pfu DNA polymerase were obtained from Thermo Fisher Scientific, Inc. (Waltham, MA, USA). Isopropyl-beta-D-thiogalactopyranoside (IPTG), chloramphenicol, N, N′-Dicyclohexylcarbodiimide (DCC), N-hydroxysuccinimide (NHS), fluorescein isothiocyanate isomer I (FITC I), Aflatoxin B1 (AFB1), Zearalanone (ZEA), Ochratoxin A (OTA), Donepezil (DON), T-2 toxin, and FB_3_ were supplied by Sigma-Aldrich (St. Louis, MO, USA). FB_1_ was obtained from Probiolab Pte. Ltd., Inc. (Singapore, Australia). FB_2_ was obtained from LKT Laboratories, Inc. (Shenzhen, China). All other chemicals and solvents were supplied by Beijing Chemical Reagent Corp, Inc. (Beijing, China). The tryptone, yeast extract, and agar powder were acquired from Oxoid, Inc. (Basingstoke, England).

### Synthesis of Fluorescent Conjugates

Briefly, 5 mg FB_1_ was dissolved in 0.5 ml methanol, followed with by triethylamine (50 μL, 7.2 mol/L) and FITC (4 mg), and then mixed well. After an overnight reaction at room temperature in the dark, small portions (50 μL) of the reaction mixture were separated by thin-layer chromatography (TLC) using a trichloromethane/methanol/acetic acid (40:10:1, v/v/v) mobile phase. The main yellow band at a R_f_ of 0.1 was scraped from the plate and extracted with 0.1 ml of methanol. FB_1_–5-DTAF was prepared in the same way as in the past research (Li et al., 2016).

### Construction of the scFv Expression Vector

The scFv gene constructed from hybridoma was generated according to our previous report (Wen et al., 2012). Briefly, the total RNA of 1  ×  10^7^ hybridoma cells was extracted by the RNeasy Mini Kit, and then cDNA was generated with the PrimeScript RT-PCR Kit. The VH and VL gene fragments were obtained by nested PCR with specific primers. Then, the VH chain and VL chain were joined with primers VLF2 and VH3 R3 with Pfu DNA polymerase (95°C 5 min; 95°C 20 s, 55°C 40 s, 72°C 2 min, 30 cycles; 72°C 10 min). Finally, the scFv gene and the expression vector pJB33 were digested with SfiI, and the digested products were linked to construct the recombinant expression vectors pJB33–4F5-scFv and pJB33–4B9-scFv.

### Expression and Purification of Soluble scFv Antibody

*Escherichia coli* (*E. coli*) RV308 was transformed with the vector pJB33–4F5-scFv and pJB33–4B95-scFv. A single bacterial colony was incubated in 2.5 ml 2 × YT medium containing chloramphenicol overnight (37°C, 200 rpm). Then, a volume of the bacterial solution was diluted 100-fold and cultured in 250 ml 2 × YT medium. When the OD_600_ value reached 0.6–0.8, IPTG was added to *E. coli* suspension to induce scFvs expression at 24°C. The system was performed at different IPTG concentrations (0, 0.25, 0.5, 0.75, 1.0, or 1.5 mM) and different incubation times (0, 2, 4, 6, 8, 10, 12 h) to optimize the scFv expression system.

Then, the *E. coli* was harvested and dissolved in 5 ml of lysis buffer. The soluble periplasm scFv in the supernatant was purified by a Ni-NTA agarose resin column according to QIAGENs’ protein purification instructions. Briefly, 50% of the Ni-NTA suspension was mixed gently with the supernatant containing the soluble periplasm scfv antibody at a ratio of 1:4 (v/v) by shaking for 2 h at room temperature. Then, the Ni-NTA agarose mixture was carefully loaded into an empty column and washed with 25 ml of washing buffer. The proteins were eluted with buffer. Subsequently, the elution was confirmed by sodium dodecyl sulfate polyacrylamide gel electrophoresis (SDS-PAGE) and western blotting. After measuring the protein concentration using the Bradford method, the proteins were aliquoted and stored at −80°C.

### Construction of FPIA Assay

The antibody dilution curve was generated by mixing 70 μL of tracer solution at the working concentration with 70 μL of two-times serially diluted scFv solution per well. Borate buffer was added to reach an overall volume of 210 μL per well. After the mixture was shaken for 10 s in a microplate reader, FP values were measured at λex 485 nm and λem 530 nm with an emission cutoff of 515 nm for FB_1_-FITC and FB_1_–5-DTAF. The sigmoidal curve was obtained by plotting the measured FP values against the concentration of FB_s_ and fitting them to a four-parameter logistic equation (Eq. 1) by the OriginPro 8.0 (Northampton, MA).

Y=(A−B)/[1+(X/C)^B]+D (1)

where A represents the responses at high asymptotes of the curve, B acts as the slope factor, C is the IC_50_ of the curve, D is the responses at low asymptotes of the curve, and X is the calibration concentration.

The concentration of FB_s_ in naturally positive maize samples was determined relative to the maize matrix-based calibration curve of FB_1_ or FB_2_ prepared in blank matrix extracts. The sensitivity of the FIPA was represented by IC_50_ values from the calibration curve. The calibration curve of FIPA for the detection of FB_1_ was constructed by OriginPro 8.0 (and data was fitted to the four-parameter logistic equation of Eq. 1).

The LOD was experimentally defined as IC_10_ from the calibration curve of FIPA (the concentration that corresponds to 10% inhibition of the maximal FP signal). The detectable range corresponds to the concentration of standard varying from IC_20_-IC_80_ from the calibration curve of FIPA.

### Cross-Reactivity Determination

To determine the specificity of this method, cross reactivities (CRs) with other mycotoxins including FB_2_, FB_3_, AFB1, ZEA, OTA, DON, and T-2 toxin were calculated by Eq. 2:

$$\text{CR}\left(\text{\%}\right)=\left({\text{IC}}_{50}\;\text{of}\;{\text{FB}}_{1}\right)/\left({\text{IC}}_{50}\;\text{of}\;\text{other}\;\text{mycotoxins}\right)\times 100\%$$ (2)

### Recovery and Precision Study

The recovery test was used to evaluate the accuracy of the developed FPIA. Briefly, blank samples were spiked with FB_s_ at three different concentrations, and then the spiked samples were submitted to FPIA for recovery analysis after pretreatment. The recovery test was carried out in three independent replicates. The samples of naturally positive maize already that were detected by high performance liquid chromatography-tandem mass spectrometry (HPLC-MS/MS) were also analyzed by the developed FPIA for the precision study.

### Homology Modeling

Submitting the gene sequences of 4F5 scFv and 4B9 scFv to the ExPASy database (https://web.expasy.org) to obtain the amino acid sequences of 4F5 scFv and 4B9 scFv. In addition, 3D structures of the 4F5 scFv and 4B9 scFv were constructed by homology modelling in the Discovery Studio 2019 software (Sharma et al., 2021). A protein-protein BLAST search in the PDB database was performed to find suitable homologous sequences (templates) of known 3D structure. Five antibody crystal structures were selected as the templates individually for 4F5 scFv and 4B9 scFv, with the identity and similarity both higher than 80%. The 3D structures of the 4F5 scFv and 4B9 scFv were then constructed by multiple aligning the heavy and light chains of these templates with the 4F5 scFv and 4B9 scFv to determine the relative spatial orientation of the VH and VL. The highest quality 3D models with the lowest probability density function energy (Ghabbour and Qabeel, 2016) and higher discrete optimized protein energy (Elizabeth and Oliver, 2012) were selected as the homology modelling of 4F5 scFv and 4B9 scFv, and then the complementarity-determining regions (CDRs) were further optimized by aligning with CDR regions of the traditional antibody sequence through the IMGT/V-QUEST database (http://www.imgt.org) to ensure the rationality of the structures. Ramachandran plot analysis was applied to evaluate the homology model (Hooft et al., 1997). Protein minimization has been carried out by Minimize tool by using Normal mode calculation methods (Ma and Karplus, 1997).

### Molecular Docking and Molecular Dynamics Simulation of scFv-FB_s_

The potential binding sites (binding pockets) of 4F5 scFv and 4B9 scFv were defined and analyzed based on a grid search and “eraser” algorithm (Liashchynskyi and Liashchynskyi, 2019; Pawar and Rohane, 2021). The binding sites were displayed as a set of points (point count) and evaluated based on the volume of each cavity calculated as the product of the number of site points and the cube of the grid spacing. Docking analysis was performed based on these predicted binding pockets by a grid-based semi-flexible molecular docking software of CDOCKER (Gagnon et al., 2014), to investigate the potential binding mechanism of FB_s_-scFv recognition. The structure of FB_1_ and FB_2_ were optimized by hybrid B3LYP functional methods in combination with the 6–31 G (d,p) basis set utilizing the GAUSSIAN 09 package (Paier et al., 2007). CDOCKER used a CHARMm-based molecular dynamics scheme to dock antigen into the antibody binding pocket. Candidate antigen poses were then created using random rigid-body rotations followed by simulated annealing. The docking results were evaluated by CDOCKER energy and Gold score fitness formula (Sadowski, 1997). To obtain a better simulation result, the best docking model with the lowest CDOCKER energy and higher fitness value was refined with a 100 ns MD simulation by GROMACS 5.0 program software in Amber14SB force field with the TIP3P solvent model (Jorgensen et al., 1983; Van Der Spoel et al., 2005; Maier et al., 2015). The solvated structure was minimized by the steepest descent method for 15,000 steps at 300 K temperature and constant pressure, the LINCS algorithm was used to constrain the bond length (Hess et al., 1997), the electrostatics interactions were calculated using a PME algorithm (Darden et al., 1993), and the hydrophobicity and interpolated charged surface area were calculated and analyzed by image-oriented modality (Cristea et al., 2011). The binding free energy of scFvs and FB_s_ was calculated by the MM/PBSA method after the MD process (Wang et al., 2019).

## Results and Discussion

### Construction, Expression, and Characterization of scFv Antibody

**FIGURE 1:**
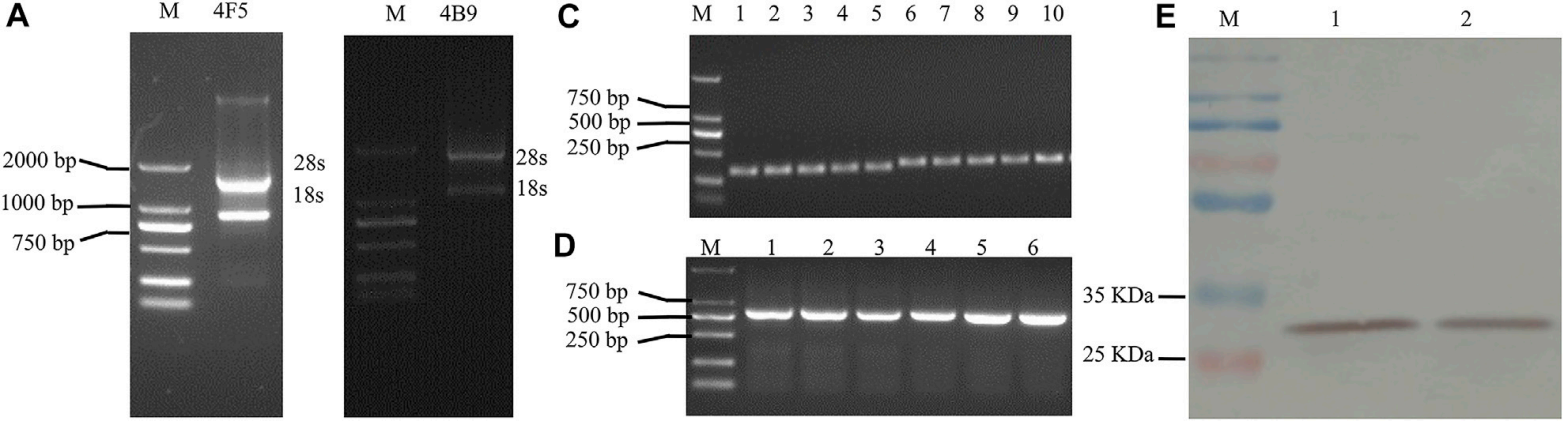
Agarose gel electrophoresis analysis and western blot analysis of 4F5 and 4B9 scFv. **(A)**: Agarose gel electrophoresis of 4F5 total RNA, M indicates DNA 2000 plus marker. **(B)**: Agarose gel electrophoresis of 4B9 total RNA, M ndicates DNA 2000 plus marker. **(C)**: Agarose gel electrophoresis of VH and VL of 4F5 and 4B9, 1–3 indicates 4F5 VH, 4–5 indicates 4B9 VH, 6–8 indicates 4F5 VL, 9–10 indicates 4B9 VL. **(D)**: Agarose gel electrophoresis of 4F5 scFv and 4B9 scFv, 1–3 indicates 4F5 scFv, 4–6 l indicates 4B9 scFv. **(E)**: The western blot analysis of 4F5 scFv and 4B9 scFv. M: marker, 1 indicates 4F5 scFv, 2 indicates 4B9 scFv.

Many researchers have demonstrated that the production of scFv antibodies from hybridoma cell lines were more effective with the native paired VH/VL than other methods. Thus, in this study, 4F5 scFv and 4B9 scFv were produced from the related hybridoma cells. Total RNA was extracted from the hybridoma cell lines (Figures 1A,B), and then the VH and VL genes were cloned from RT-PCR cDNA with specific primers (Supplementary Table S1). The VH gene fragments of 4F5 and 4B9 hybridomas cells were both 339 bp, and VL gene fragments were 321 and 330 bp, respectively (Figure 1C). Then, the VH and VL genes were linked by a peptide to construct the scFv gene (VH-(Gly_4_Ser)_3_-VL) with the gene splicing performed by overlap extension PCR (SOE-PCR), which was expected to be approximately 700–750 bp. As shown in Figure 1D, the results of agarose gel electrophoresis analysis showed that the scFv genes were approximately 750 bp, which were consistent with the anticipated length (Supplementary Table S2). Then the scFv genes were transformed into expression vector pJB33 for effective recombinant expression.

*E. coli* host system is widely regarded as the most suitable host for the expression of recombinant antibody fragments with the advantages of faster growth and easier genetic manipulation. However, the soluble expression of scFv still needs to be improved (Ahmad et al., 2012). Choi et al. have tried to clone the scFv into these vectors as pET-29b (+), pET-26b (+), and pGEX-5X-3 vectors but only expressed some inactive inclusion body proteins without any binding activity (Choi et al., 2004), which show that the pET series vectors are not suitable for all scFv expression. pJB33 was another effective prokaryotic expression with the advantage of strong periplasmic expression via its specific ribosome binding site (Schaefer and Plückthun, 2010), which was indicated to be more suitable for recombinant antibody expression and could also improve the expression efficiency of recombinant antibodies compared with that of other expression vectors (Krebber et al., 1997). Wen et al. successfully constructed a broad-spectrum fluoroquinolones (FQ_s_)-scFv using pJB33 vectors, and Chen et al. successfully produced bispecific antibodies to FQ_s_ and sulfonamides by constructing two scFv sequences into the pJB33 vector (Wen et al., 2012; Chen et al., 2014).

In this study, pJB33 was used as the expression vector of 4B9 and 4F5 scFv. The expression system was optimized with 0.25 mM IPTG to induce the expression of scFvs in the periplasmic cavity as soluble protein at 20°C for 16 h (Figure 1E and Supplementary Figure S1). Then, scFvs were purified by a Ni-NTA agarose resin column using a 6 × His-tag in the scFv. The western blotting result of the purified scFvs showed a single band at approximately 30 kD that was consistent with the molecular weight of total expected scFv, c-Myc label, and 6 × His-tag label, which indicated that no degradation occurred and no dimers were present in the process of antibody expression and extraction.

In theory, the recognition ability of a scFv should be similar to its parental mAbs because scFv contains the key binding region of the variable domain of the light and heavy chains. However, the total RNA extracted from hybridoma cells contains a number of nonspecific light and heavy chain mRNAs (Toleikis and Frenzel, 2012), and PCR also introduces some errors. It is often difficult to obtain scFvs that can maintain the same specificity as the maternal mAbs. Some previous articles reported that scFv antibodies showed similar or better performances than that of the parental mAbs (Wen et al., 2012), but other articles reported that scFv antibodies performed worse than the parental mAbs (Xie et al., 2018). Min et al. amplified the anti-FB_1_ single-stranded antibody gene on the basis of anti-FB_1_ hybridoma cells and converted it to *E. coli* to obtain anti-FB_1_ single-stranded antibody with the an IC_50_ reducing 12 times approximately 2000 ng/ml compared with the original mAb (Min et al., 2010). In this study, two scFvs of 4B9 and 4F5 were produced based on a hybridoma cell lines against FB_s_. The indirect competitive ELISA (icELISA) results showed that the affinity of 4F5 scFv with FB_1_ (IC_50_ = 12.99 ng/ml) was lower than that of mAb (IC_50_ = 6.3 ng/ml), whereas the affinity of 4B9 scFv with FB_1_ (IC_50_ = 48.61 ng/ml) was higher than that of 4B9 mAb (IC_50_ = 85.1 ng/ml) (Supplementary Figure S2). The above results indicated that the 4F5 scFv and 4B9 scFv could be applied to the detection of FB_s_ in the later immunoassay experiment.

### Development of FPIA in Maize

#### Development of FPIA

ScFv, a small engineered antibody, has been indicated as a possible replacement of the mAbs in detection and diagnosis (Wang et al., 2007). ScFvs have been applied mostly to small molecule detection in ELISA, chemiluminescent enzyme immunoassay (CLISA) (Dong et al., 2021; Wang et al., 2014), fluorescence resonance energy transfer (FRET) (Lee et al., 2015), chemiluminescent resonance energy transfer (CRET) immunoassay (Dou et al., 2021), and so on. In this study, a FPIA was firstly constructed with the anti-FB_s_ scFv.

Different tracers have been observed to perform differently when the same mAb is used (Maragos et al., 2001). In this study, two tracers (FB_1_-FITC, FB_1_–5-DTAF) were synthesized to achieve the most sensitive FPIA (Supplementary Figure S3 and Supplementary Table S3). The tracer concentration greatly influenced the sensitivity of the competitive FPIA, and a higher sensitivity in the method was always accompanied by a lower tracer concentration, which caused the detection signal to become instable (Smith and Eremin, 2008). In this method, when FI was approximately 20 times higher than that of the background, these concentrations of tracers were selected as the working concentration with the most stable fluorescence signal value (the SD of the FP values of both free and bound tracer was less than 5 mP).

**FIGURE 2:**
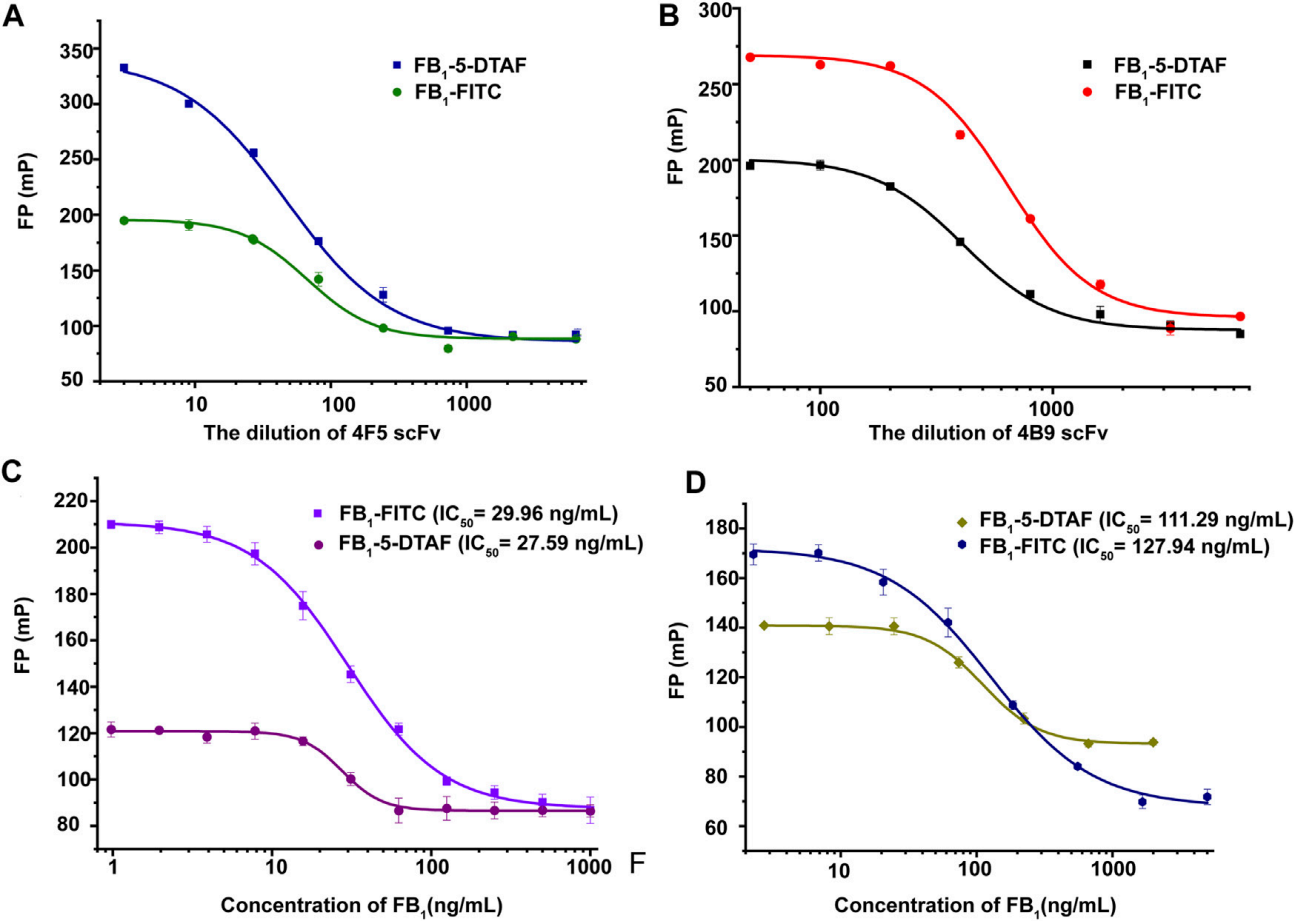
Optimization of scFv and tracer concentrations. **(A)**: Optimization of 4F5 scFv concentration. **(B)**: Optimization of 4B9 scFv concentration. **(C)**: Optimization of the tracer concentrations of with 4F5 scFv. **(D)**: Optimization of the tracer concentrations with 4B9 scFv.

The working concentration of the antibody was another important parameter that affected the sensitivity of FPIA. The FPIA method established by Sheng et al. selected the antibody concentration corresponded to 75% fluorescent tracer binding with the best sensitivity (Sheng et al., 2014). In this study, the scFv dilution that corresponded to 70% tracer binding was chosen as the optimum concentration to obtain a wider analytical range according to previous studies (Deryabina et al., 2005). The best antibody dilutions for FB_1_-FITC with 4F5 scFv and 4B9 scFv were 1/30 and 1/700, for FB_1_–5-DTAF with 4F5 scFv and 4B9 scFv were 1/50 and 1/400 in the maize matrix (Figures 2A,B). Calibration curves in a blank maize matrix was conducted under the optimal conditions. The IC_50_ of FB_1_-FITC was lower than FB_1_–5-DTAF, but the detection range of FB_1_-FITC was larger and gave the widest assay window (δ FP, FP_max_–FP_min_) and lowest background. Thus, the FB_1_-FITC was chosen as the tracer of 4B5 and 4F9 scFv (Figures 2C,D).

**FIGURE 3:**
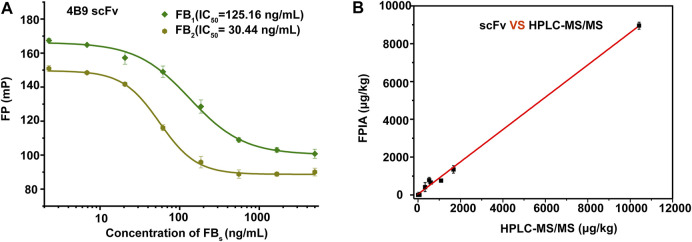
Matrix effect and precise analysis of the FIPA. **(A)**: Calibration curves in a blank maize matrix of FPIA with 4B9 scFv. **(B)**: Correlation analysis between HPLC–MS/MS and the developed FPIA (*n* = 3).

The specificity of scFv was evaluated by CRs with other mycotoxins (FB_2_, FB_3_, AFB_1_, ZEA, OTA, DON, and T-2 toxin) by FPIA (Table 1). The results showed negligible CRs of both 4F5 scFv and 4B9 scFv with AFB_1_, ZEA, OTA, DON, and T-2 toxin. The cross reactivity with FB_2_ (420.16%) of 4B9 scFv in maize matrix was higher than that with FB_2_ compared with 4F5 scFv (2.02%). Thus, FB_1_-FITC and 4B9 scFv pair was utilized to detect FB_1_ and FB_2_ in maize in FPIA with the LOD of 441.54 μg/kg and 344.933 μg/kg, respectively (Figure 3A). Li et al. also used mAb 4B9 for detection of FB_1_ and FB_2_ in maize due to its high CR with FB_2_ (98.9% in maize matrix) (Li et al., 2015).

**TABLE 1:** The detection parameters of 8 mycotoxins by 4F5 and 4B9 scFv.

Mycotoxin	Structure	scFv			
		4B9		4F5	
—	—	IC_50_ (ng ml^−1^)	CR (%)	IC_50_ (ng ml^−1^)	CR (%)
FB_1_ ^b^	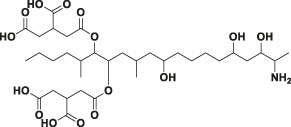	125.16	100.0	29.36	100
FB_2_ ^c^	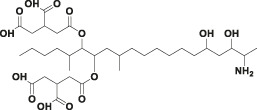	30.44	420.16	1477.82	2.02
FB_3_ ^d^	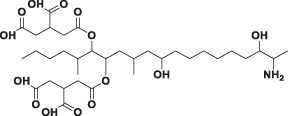	147.94	85.19	62.93	47.61
AFB_1_ ^e^	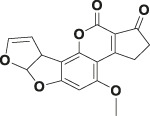	- ^a^	<1%	- ^a^	<0.1%
ZEA^f^	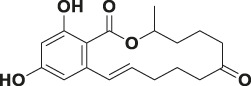	- ^a^	<1%	- ^a^	<0.1%
OTA^g^	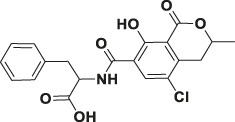	- ^a^	<1%	- ^a^	<0.1%
DON^h^	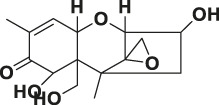	- ^a^	<1%	- ^a^	<0.1%
T-2 toxin^i^	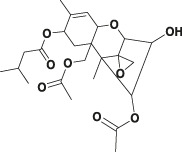	- ^a^	<1%	- ^a^	<0.1%

a Note: means IC_50_ > 10,000 ng mL^−1^.

b indicates the Fumonisin B_1_ ((2S,2′S)-2,2′-[(5S,6,7,9R,11S,16R,18S,19S)-19-Amino-11,16,18-trihydroxy-5,9-dimethylicosane-6,7-diyl]bis[oxy(2-oxoethane-2,1-diyl)]dibutanedioic acid).

c indicates the Fumonisin B_2_ ((2R,2′R)-{[(5R,6R,7S,9S,16R,18S,19S)-19-Amino-16,18-dihydroxy-5,9-dimethylicosane-6,7-diyl]bis[oxy(2-oxoethane-2,1-diyl)]}dibutanedioic acid).

d indicates the Fumonisin B_3_ (2-[2-[(5R,6R,7S,9S,11R,18R,19S)-19-amino-6-(3,4-dicarboxybutanoyloxy)-11,18-dihydroxy-5,9-dimethylicosan-7-yl]oxy-2-oxoethyl]butanedioic acid).

e indicates the Aflatoxin B1 (2,3,6aR,9aS-tetrahydro-4-methoxy-1H,11H-cyclopenta[c]furo[3′,2':4,5]furo[2,3-h][1]benzopyran-1,11-dione).

f indicates the Zearalanone ((3S)-3,4,5,6,9,10,11,12-octahydro-14,16-dihydroxy-3-methyl-1H-2-benzoxacyclotetradecin-1,7(8H)-dione).

g indicates the Ochratoxin A (N-[[(3R)-5-chloro-3, 4-dihydro-8-hydroxy-3-methyl-1-oxo-1H-2-benzopyran-7-yl]carbonyl]-L-phenylalanine).

h indicates the Donepezil (2,3-dihydro-5,6-dimethoxy-2[[1-(phenylmethyl)-4-piperidinyl]methyl]-1H-inden-1-one).

i indicates the T-2 Toxin ((3α,4β,8α)-12,13-epoxy-4,15-diacetate 8-(3-methylbutanoate) trichothec-9-ene-3,4,8,15-tetrol).

#### Recovery and Precision Study

As a homogeneous assay, FPIA is more susceptible to be interfered by matrix effects than other heterogeneous assays. Therefore, the investigation of matrix effect is an important part of FPIA. In this study, methanol/water (2:3, v/v) was used to extract FB_s_ from maize samples according to a previous report (Li et al., 2015). A maize matrix-based calibration curve was used to determine the concentration of FBs in naturally contaminated maize samples to reduce the background interference of maize extracts. In spiked maize samples, FB_1_ and FB_2_ were separately added at 300, 500, and 1,000 μg/kg, and the recovery of 4B9 scFv ranged from 88.9 to 104.1% and the CVs were 2.3–14.1% (Table 2). This corresponded to a previous report that the recovery of 4B9 mAb with FB_1_ or FB_2_ were 84.7–93.6% and the CVs were less than 9.9% (Li et al., 2015).

**TABLE 2:** Detection of FB_1_、FB_2_ in spiked maize samples (*n* = 3).

Fumonisins	Tracer	Antibody	Spiked concentration (μg kg-1)	Recovery rate (%)	CV (%)
FB_1_	FB_1_-FITC	4B9 mAb	2,000	89.2	6.1
			1,000	89.9	4.0
			500	93.6	3.9
FB_2_			2,000	87.3	3.2
			1,000	85.4	7.6
			500	84.7	9.9
FB_1_		4B9 scfv	2,000	88.9	9.3
			1,000	99.7	9.8
			500	89.68	11.7
FB_2_			2,000	92.4	9.1
			1,000	104.1	2.3
			500	92.3	14.1

Recently reported immunoassays for FB_1_ were mostly based on polyclonal and monoclonal antibodies. A rapid immunochromatographic test strip had been developed for detection of FB_s_ based on the mAb with LOD of 60 ng/ml for FB_1_ and cross reactivities with FB_2_ and FB_3_ of 385 and 72.4% (Yao et al., 2017). Compared with mAb, scFv, as the smallest functional unit of antibody, have been applied in many studies. Zou et al. established an icELISA based on anti-FB_1_ scFv only for FB_1_ detection with the IC_50_ of 12.67 ng/ml, without CR with other mycotoxins (Zou et al., 2014). In this study, we developed a homogeneous assay of FPIA based on scFv for detection of FB_1_ and FB_2_ in positive maize samples, and results showed that the FPIA method was in good consistency with HPLC-MS/MS (Figure 3B and Supplementary Table S5), which indicated that the established method had high reliability and also demonstrated the feasibility of the scFv to replace the core part of mAbs and pAbs in many immunoassay technologies for small molecular detection.

### Molecular Interactions of scFvs and FB_s_

#### Homology Modeling of scFv

**FIGURE 4:**
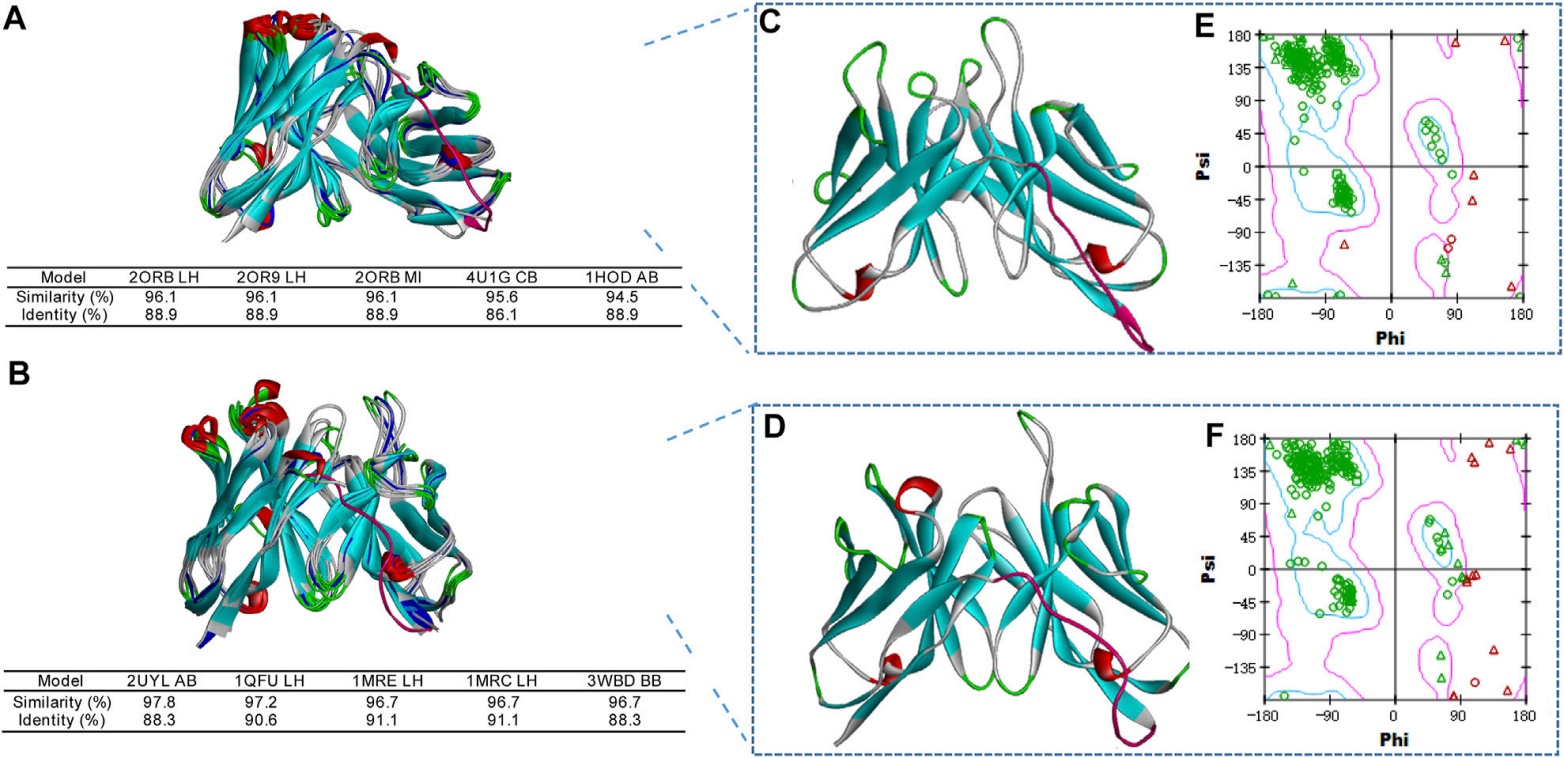
Homology modelling of 4F5 scFv and 4B9 scFv. **(A,B)**: The multiple alignment of the five templates with 4F5 scFv and 4B9 scFv. **(C,D)**: The 3D models of 4F5 scFv and 4B9 scFv. **(E,F)**: The Ramachandran plots of 4F5 scFv and 4B9 scFv.

In this study, the 3D structures of 4F5 scFv and 4B9 scFv were predicted by the homology modelling, combining the analysis of its amino acid distribution in Supplementary Tables S6, S7. Five antibody crystal structures with the higher similarity (>85.4%) and greater identity (>76%) were chosen based on the top performing homology modelling algorithm, MODELER (Parveen et al., 2019), for multiple alignment analysis with the 4F5 scFv and 4B9 scFv (Figures 4A,B). Then the 3D models of 4F5 scFv and 4B9 scFv were constructed based on the multiple alignment results and the CDR regions were further optimized based on the IMGT/V-QUEST database (http://www.imgt.org), which composed the typical antiparallel β-sheets and loops in variable regions of scFv (Figures 4C,D). Ramachandran plot was applied to verify the predicted torsion angles in the 3D models and evaluate the fitness of the protein sequence to ensure the rationality, which showed that the constructed model of 4F5 scFv and 4B9 scFv with 95.4 and 97.4% domain amino acid residues locating in the allowed region respectively (Figures 4E,F), which met the requirement of a good quality model with over 90% of residues in the allowed region (Rodriguez et al., 1998); meanwhile, the residues in the disallowed region also do not belong to the CDR regions. The result indicated that all residues in the model were valid and the 3D structure constructed was reasonable.

**FIGURE 5:**
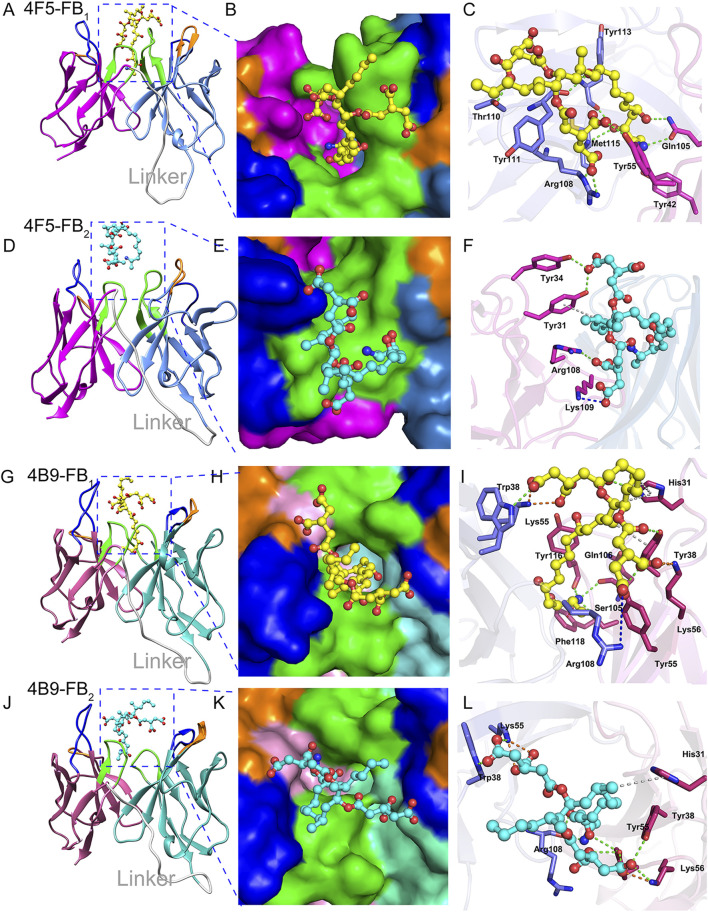
Interaction analysis of scFvs-FB_s_. **(A,D)**: The 4F5-FB_1_ complex and 4F5-FB_2_ complex, marine indicates VL, magenta indicates VH, gray indicates linker, blue indicates CDR1, orange indicates CDR2, green indicates CDR3. **(B,E)**: The FB_1_ in the binding pocket of 4F5 scFv and FB_2_ in the binding pocket of 4F5 scFv. **(C,F)**: The interactions between FB_1_ and 4F5 scFv, and FB_2_ and 4F5; green dashed line indicates the hydrogen bond interaction, blue dashed line indicates the electrostatic attraction, gray indicates the Pi-Alkyl, and orange indicates the salt bridge. **(G,J)**: The 4B9-FB_1_ complex and 4B9-FB_2_ complex; hot pink indicates VL, cyan indicates VH, gray indicates linker, blue indicates CDR1, orange indicates CDR2, green indicates CDR3. **(H,K)**: FB_1_ in the binding pocket of 4B9 scFv and FB_2_ in the binding pocket of 4B9 scFv. **(I,L)**: The interactions between FB_1_ and 4B9 scFv, and FB_2_ and 4B9; green dashed line indicates the hydrogen bond interaction, blue dashed line indicates the electrostatic attraction of pi-cation and attractive charge, gray indicates the pi-Alkyl, and orange indicates the salt bridge.

Molecular docking is one of the important methods to reveal the types and distances of forces in different antigen-antibody interaction (Liu et al., 2016). After the docking, the scFv-FBs complexes with the lowest CDOCKER energy and higher fitness value were obtained, and a 100 ns MD simulation was performed for the scFv-FBs to get a stable combination complex. The root mean square deviation (RMSD) values of the backbone atoms were calculated to monitor the structural stability of the complexes (Supplementary Figure S4). After optimization of the molecular dynamic simulation, intermolecular interactions were shown in Figure 5 and Supplementary Table S8. During the immune process, the carboxyl terminus of FBs was coupled to the protein by the carbodiimide method to prepare the immunogen, and thus amino terminus was the main antibody recognition site due to the effect of steric hindrance. Thus, the simulation result showed that the amino terminus of FB1 and FB2 inserted into the binding pocket, and fitted well with narrow cavity formed by the 4B9 scFv CDR regions of L1, L2, L3, H1, and H3 (Figures 5G,H,J,K) with the large contacted surface (Supplementary Figures S5C,D). But in 4F5 scFv, only the FB1 stably inserted in the binding pocket formed by CDR H1, CDR H3, CDR L2, and CDR L3 (Figures 5A,B); FB2 did not fit the pocket well with the only binding with CDR L1 and CDRL3 and with a small contacted surface (Supplementary Figures S5A,B), which showed weak interaction with 4F5 scFv (Figures 5D,E). The scFv formed quite different contact surface with different size, charged properties, and hydrophobicity with different CDRs in scFv may probably account for their different interaction, shown in the Supplementary Figures S5, S6.

The difference between FB_1_ and FB_2_ structure is only the hydroxyl in the C10 position (Supplementary Figure S7); the key hydrogen bonds of FB_1_ and 4F5 scFv were exactly between the O3 in this hydroxyl oxygen and residue Tyr113 of CDR H3, and also other hydrogen bonds in Figure 5C and Supplementary Table S8 which lead to a stable binding of FB_1_ with 4F5 scFv. The lack of this hydroxyl group in FB_2_ led to a poor adaptation to 4F5 scFv binding pocket and further affected the recognition performance (Figure 5F). Unlike 4F5 scFv, 4B9 scFv dramatically formed both strong hydrogen bond interactions with the amino in FB_1_ and FB_2_. The Ser 105 and Ser 106 formed two hydrogen bonds H104 and H105 in the amino of FB_1_ and also the Arg108 formed two hydrogen bonds with H103 and H104 in the amino of FB_2_, and some electrostatic forces of attractive charge, salt bridge and were also formed between the charged groups of positively charged amino/the negatively charged hydroxyl with the Lys, Arg, and His (Supplementary Table S8). Thus, both FB_1_ and FB_2_ stably inserted into the narrow binding pocket of 4B9 scFv (Figures 5I,L).

Above all, the 4B9 scFv mainly recognized the amino of the FB_1_ and FB_2_ while the 4F5 was more inclined to the hydroxyl in C10, which lead to different recognition for FBs and scFv. The binding free energy of FB_2_ and 4B9 scFv (−31.57 kcal/mol) was also lower than that of 4F5 scFv (−19.74 kcal/mol), which could explain the different IC_50_ value of FB_2_ with 4F5 scFv (1,378.22 ng/ml) and 4B9 scFv (30.44 ng/ml). The above research provided us new information to better understand the FB_s_-antibody recognition mechanism, which will guide future design and antibody improvement.

## Conclusion

In this study, scfvs were successfully constructed based on 4F5 and 4B9 hybridoma cells. Then, the optimized FPIA method was established for simultaneously detecting the FB_1_ and FB_2_ in maize by 4B9 scfv with the recoveries of 88.9–104.1% with the CVs <14.1%. The molecular recognition mechanism for scfv-FBs was furthermore studied by homology modeling, molecular docking, and MD simulation method, and showed that 4B9 scfv had a stronger binding with FB_2_ compared with 4F5 scfv, and also the CDRs of 4B9 scfv formed a narrow cavity that could encapsulate almost the entire FB_2_ molecule compared with 4F5 scfv. The different binding conformations of 4F5 scfv, 4B9 scfv with FB_2_ molecules explained the different affinity performance. Above all, this study provided a good demonstration for the further study of molecular interactions between antibody and antigen, and also supplied a reasonable guidance to the antibody production and immunoassay development.

## Data Availability

The original contributions presented in the study are included in the article/Supplementary Material, and further inquiries can be directed to the corresponding author.

## References

[B1] Ahmad Z. A. Yeap S. K. Ali A. M. Ho W. Y. Alitheen N. B. M. Hamid M. (2012). scFv Antibody: Principles and Clinical Application. Clin. Dev. Immunol. 2012, 980250. 10.1155/2012/980250 22474489 PMC3312285

[B2] Arata Y. (1991). Nuclear Magnetic Resonance Studies of Antibody‐Antigen Interactions. Ciba Found. Symp. 159, 40–54. 1959452

[B55] Chen M. Wen K. Tao X. Xie J. Wang L. Li Y. (2014). Cloning, Expression, Purification and Characterization of a Bispecific Single-Chain Diabody Against Fluoroquinolones and Sulfonamides in Escherichia coli. Protein Expr. Purif. 100, 19–25. 10.1016/j.pep.2014.04.015 24816423

[B3] Choi G.-H. Lee D.-H. Min W.-K. Cho Y.-J. Kweon D.-H. Son D.-H. (2004). Cloning, Expression, and Characterization of Single-Chain Variable Fragment Antibody against Mycotoxin Deoxynivalenol in Recombinant *Escherichia C* . Protein Expr. Purif. 35 (1), 84–92. 10.1016/j.pep.2003.12.008 15039070

[B4] Cristea P. D. Arsene O. Tuduce R. Nicolau D. V. (2011). “Protein Surface Analysis. Part 1: Hydrophobicity Densities,” in ISSCS 2011-International Symposium on Signals, Circuits and Systems (IEEE), PP: 1–4. 10.1109/ISSCS.2011.5978748

[B5] Darden T. York D. Pedersen L. (1993). Particle Mesh Ewald: AnN⋅Log(N) Method for Ewald Sums in Large Systems. J. Chem. Phys. 98 (12), 10089–10092. 10.1063/1.464397

[B6] Deryabina M. A. Yakovleva Y. N. Popova V. A. Eremin S. A. (2005). Determination of the Herbicide Acetochlor by Fluorescence Polarization Immunoassay. J. Anal. Chem. 60 (1), 80–85. 10.1007/s10809-005-0063-4

[B7] Dong J. Li Z. Wang Y. Jin M. Shen Y. Xu Z. (2021). Generation of Functional Single-Chain Fragment Variable from Hybridoma and Development of Chemiluminescence Enzyme Immunoassay for Determination of Total Malachite green in tilapia Fish. Food Chem. 337, 127780. 10.1016/j.foodchem.2020.127780 32799164 PMC7541715

[B8] Dou L. Pan Y. Ma M. Zhang S. Shen J. Wang Z. (2021). Antibody Engineering-Driven Controllable Chemiluminescence Resonance Energy Transfer for Immunoassay with Tunable Dynamic Range. Analytica Chim. Acta 1152, 338231. 10.1016/j.aca.2021.338231 33648650

[B9] Elizabeth H. Oliver F. (2012). Evaluation and Optimization of Discrete State Models of Protein Folding. J. Phys. Chem. 116 (37), 11405–11413. 10.1021/jp3044303 22958200

[B10] Gagnon J. K. Law S. M. Brooks C. L. (2014). Flexible CDOCKER: Development and Application of a Pseudo‐Explicit Structure‐Based Docking Method within CHARMM. J. Comput. Chem. 106 (2), 646a. 10.1016/j.bpj.2013.11.3576 PMC4776757 26691274

[B11] Ghabbour H. A. Qabeel M. M. (2016). Synthesis, Crystal Structure, Density Function Theory, Molecular Docking and Antimicrobial Studies of 2-(3-(4- Phenylpiperazin-1-Yl) Propyl) Isoindoline-1,3-Dione. Trop. J. Pharm. Res. 15 (2), 385–392. 10.4314/tjpr.v15i2.23

[B12] Hess B. Bekker H. Berendsen H. J. C. Fraaije J. G. E. M. (1997). LINCS: A Linear Constraint Solver for Molecular Simulations. J. Comput. Chem. 18 (12), 1463–1472. 10.1002/(sici)1096-987x(199709)18:12<1463::aid-jcc4>3.0.co;2-h

[B13] Hooft R. W. W. Sander C. Vriend G. (1997). Objectively Judging the Quality of a Protein Structure from a Ramachandran Plot. Bioinformatics 13 (4), 425–430. 10.1093/bioinformatics/13.4.425 9283757

[B14] Hu Z.-Q. Li H.-P. Liu J.-L. Xue S. Gong A.-D. Zhang J.-B. (2016). Production of a Phage-Displayed Mouse ScFv Antibody against Fumonisin B1 and Molecular Docking Analysis of Their Interactions. Biotechnol. Bioproc. E 21 (1), 134–143. 10.1007/s12257-015-0495-0

[B15] Jorgensen W. L. Chandrasekhar J. Madura J. D. Impey R. W. Klein M. L. (1983). Comparison of Simple Potential Functions for Simulating Liquid Water. J. Chem. Phys. 79 (2), 926–935. 10.1063/1.445869

[B16] Krebber A. Bornhauser S. Burmester J. Honegger A. Willuda J. Bosshard H. R. (1997). Reliable Cloning of Functional Antibody Variable Domains from Hybridomas and Spleen Cell Repertoires Employing a Reengineered Phage Display System. J. immunological Methods 201 (1), 35–55. 10.1016/s0022-1759(96)00208-6 9032408

[B17] Lee J. Brennan M. B. Wilton R. Rowland C. E. Rozhkova E. A. Forrester S. (2015). Fast, Ratiometric FRET from Quantum Dot Conjugated Stabilized Single Chain Variable Fragments for Quantitative Botulinum Neurotoxin Sensing. Nano Lett. 15 (10), 7161–7167. 10.1021/acs.nanolett.5b03442 26397120

[B18] Li C. Mi T. Oliveri Conti G. Yu Q. Wen K. Shen J. (2015). Development of a Screening Fluorescence Polarization Immunoassay for the Simultaneous Detection of Fumonisins B1 and B2 in maize. J. Agric. Food Chem. 63 (20), 4940–4946. 10.1021/acs.jafc.5b01845 25942573

[B19] Li C. Wen K. Mi T. Zhang X. Zhang H. Zhang S. (2016). A Universal Multi-Wavelength Fluorescence Polarization Immunoassay for Multiplexed Detection of Mycotoxins in Maize. Biosens. Bioelectron. 79, 258–265. 10.1016/j.bios.2015.12.033 26720917

[B20] Liashchynskyi P. Liashchynskyi P. (2019). Grid Search, Random Search, Genetic Algorithm: A Big Comparison for NAS. arXiv preprint arXiv:1912.06059.

[B21] Liu J. Zhang H. C. Duan C. F. Dong J. Zhao G. X. Wang J. P. (2016). Production of Anti-Amoxicillin ScFv Antibody and Simulation Studying its Molecular Recognition Mechanism for Penicillins. J. Environ. Sci. Health B 51 (11), 742–750. 10.1080/03601234.2016.1198639 27383141

[B22] Liu Y. H. Guo Y. R. Wang C. M. Gui W. J. Zhu G. N. (2010). Homology Modeling of Anti-Parathion Antibody and its Interaction with Organophosphorous Pesticides and Analogues. J. Environ. Sci. Health Part B 45 (8), 819–827. 10.1080/03601234.2010.515501 20972920

[B23] Ma J. Karplus M. (1997). Ligand-Induced Conformational Changes in Ras P21: A Normal Mode and Energy Minimization Analysis. J. Mol. Biol. 274 (1), 114–131. 10.1006/jmbi.1997.1313 9398520

[B24] Mahalakshmi N. Ravishankaran R. Kamatchi R. Sangith N. Kaliraj P. Meenakshisundaram S. (2019). Molecular Evolution of Single Chain Fragment Variable (scFv) for Diagnosis of Lymphatic Filariasis. Mol. Biol. Rep. 46 (5), 5409–5418. 10.1007/s11033-019-04995-1 31512046

[B25] Maier J. A. Martinez C. Kasavajhala K. Wickstrom L. Hauser K. E. Simmerling C. (2015). ff14SB: Improving the Accuracy of Protein Side Chain and Backbone Parameters from ff99SB. J. Chem. Theor. Comput. 11 (8), 3696–3713. 10.1021/acs.jctc.5b00255 PMC4821407 26574453

[B26] Maragos C. M. Jolley M. E. Plattner R. D. Nasir M. S. (2001). Fluorescence Polarization as a Means for Determination of Fumonisins in maize. J. Agric. Food Chem. 49 (2), 596–602. 10.1021/jf0010782 11261998

[B27] Min W.-K. Cho Y.-J. Park J.-B. Bae Y.-H. Kim E.-J. Park K. (2010). Production and Characterization of Monoclonal Antibody and its Recombinant Single Chain Variable Fragment Specific for a Food-Born Mycotoxin, Fumonisin B1. Bioproc. Biosyst Eng 33 (1), 109–115. 10.1007/s00449-009-0350-9 19597742

[B28] Nencetti S. Mazzoni M. R. Ortore G. Lapucci A. Giuntini J. Orlandini E. (2011). Synthesis, Molecular Docking and Binding Studies of Selective Serotonin Transporter Inhibitors. Eur. J. Med. Chem. 46 (3), 825–834. 10.1016/j.ejmech.2010.12.018 21272963

[B29] Paier J. Marsman M. Kresse G. (2007). Why Does the B3LYP Hybrid Functional Fail for Metals? J. Chem. Phys. 127 (2), 024103. 10.1063/1.2747249 17640115

[B30] Parveen T. Kamran M. Fatmi M. Q. (2019). Structural and Dynamical Thermostability of Psychrophilic Enzyme at Various Temperatures: Molecular Dynamics Simulations of Tryptophan Synthase. Arch. Biochem. Biophys. 663, 297–305. 10.1016/j.abb.2019.01.022 30703344

[B31] Pawar S. S. Rohane S. H. (2021). Review on Discovery Studio: An Important Tool for Molecular Docking. Asian J. Res. Chem. 14 (1), 86–88. 10.5958/0974-4150.2021.00014.6

[B32] Rodriguez R. Chinea G. Lopez N. Pons T. Vriend G. (1998). Homology Modeling, Model and Software Evaluation: Three Related Resources. Bioinformatics 14 (6), 523–528. 10.1093/bioinformatics/14.6.523 9694991

[B33] Sadowski J. (1997). A Hybrid Approach for Addressing Ring Flexibility in 3D Database Searching. J. Computer-Aided Mol. Des. 11 (1), 53–60. 10.1023/a:1008023427310 9139112

[B34] Schaefer J. V. Plückthun A. (2010). “Improving Expression of scFv Fragments by Co-expression of Periplasmic Chaperones,” in Antibody Engineering. Editors Kontermann R. Dübel S (Berlin, Heidelberg: Springer), 345–361. 10.1007/978-3-642-01147-4_27

[B35] Sharma S. Sharma A. Gupta U. (2021). Molecular Docking Studies on the Anti-Fungal Activity of Allium Sativum (Garlic) against Mucormycosis (Black Fungus) by BIOVIA Discovery Studio Visualizer 21.1. 0.0. Ann. Antivir. Antiretrovir 5 (1), 028–032. 10.17352/aaa.000013

[B36] Sheng Y. J. Eremin S. Mi T. J. Zhang S. X. Shen J. Z. Wang Z. H. (2014). The Development of a Fluorescence Polarization Immunoassay for Aflatoxin Detection. Biomed. Environ. Sci. 27 (2), 126–129. 10.3967/bes2014.027 24625404

[B37] Sheng Y. Jiang W. De Saeger S. Shen J. Zhang S. Wang Z. (2012). Development of a Sensitive Enzyme-Linked Immunosorbent Assay for the Detection of Fumonisin B1 in maize. Toxicon 60 (7), 1245–1250. 10.1016/j.toxicon.2012.08.011 22960014

[B38] Smith D. S. Eremin S. A. (2008). Fluorescence Polarization Immunoassays and Related Methods for Simple, High-Throughput Screening of Small Molecules. Anal. Bioanal. Chem. 391 (5), 1499–1507. 10.1007/s00216-008-1897-z 18264817

[B39] Songsermsakul P. Razzazi-Fazeli E. (2008). A Review of Recent Trends in Applications of Liquid Chromatography-Mass Spectrometry for Determination of Mycotoxins. J. Liquid Chromatogr. Relat. Tech. 31 (11-12), 1641–1686. 10.1080/10826070802126395

[B40] Srivastava A. Nagai T. Srivastava A. Miyashita O. Tama F. (2018). Role of Computational Methods in Going beyond X-ray Crystallography to Explore Protein Structure and Dynamics. Int. J. Mol. Sci. 19 (11), 3401. 10.3390/ijms19113401 PMC6274748 30380757

[B41] Toleikis L. Frenzel A. (2012). “Cloning Single-Chain Antibody Fragments (ScFv) from Hyrbidoma Cells,” in Antibody Engineering. Editor Chames P. (Totowa, NJ: Humana Press), 59–71. 10.1007/978-1-61779-974-7_3 22907345

[B42] Van Der Spoel D. Lindahl E. Hess B. Groenhof G. Mark A. E. Berendsen H. J. C. (2005). GROMACS: Fast, Flexible, and Free. J. Comput. Chem. 26 (16), 1701–1718. 10.1002/jcc.20291 16211538

[B43] Wang E. Sun H. Wang J. Wang Z. Liu H. Zhang J. Z. H. (2019). End-Point Binding Free Energy Calculation with MM/PBSA and MM/GBSA: Strategies and Applications in Drug Design. Chem. Rev. 119 (16), 9478–9508. 10.1021/acs.chemrev.9b00055 31244000

[B44] Wang Z. Li H. Li C. Yu Q. Shen J. De Saeger S. (2014). Development and Application of a Quantitative Fluorescence-Based Immunochromatographic Assay for Fumonisin B1 in maize. J. Agric. Food Chem. 62 (27), 6294–6298. 10.1021/jf5017219 24930671

[B45] Wang Z. Zhang S. Nesterenko I. S. Eremin S. A. Shen J. (2007). Monoclonal Antibody-Based Fluorescence Polarization Immunoassay for Sulfamethoxypyridazine and Sulfachloropyridazine. J. Agric. Food Chem. 55 (17), 6871–6878. 10.1021/jf070948d 17661485

[B46] Weidenbörner M. (2001). Foods and Fumonisins. Eur. Food Res. Technol. 212 (3), 262–273. 10.1007/s002170000259

[B47] Wen K. Nölke G. Schillberg S. Wang Z. Zhang S. Wu C. (2012). Improved Fluoroquinolone Detection in ELISA through Engineering of a Broad-Specific Single-Chain Variable Fragment Binding Simultaneously to 20 Fluoroquinolones. Anal. Bioanal. Chem. 403 (9), 2771–2783. 10.1007/s00216-012-6062-z 22549819

[B48] Xie S. Wen K. Xie J. Zheng Y. Peng T. Wang J. (2018). Magnetic-Assisted Biotinylated Single-Chain Variable Fragment Antibody-Based Immunoassay for Amantadine Detection in Chicken. Anal. Bioanal. Chem. 410 (24), 6197–6205. 10.1007/s00216-018-1227-z 30006725

[B49] Yao J. Sun Y. Li Q. Wang F. Teng M. Yang Y. (2017). Colloidal Gold-McAb Probe-Based Rapid Immunoassay Strip for Simultaneous Detection of Fumonisins in maize. J. Sci. Food Agric. 97 (7), 2223–2229. 10.1002/jsfa.8032 27616272

[B50] Zhang X. Eremin S. A. Wen K. Yu X. Li C. Ke Y. (2017). Fluorescence Polarization Immunoassay Based on a New Monoclonal Antibody for the Detection of the Zearalenone Class of Mycotoxins in maize. J. Agric. Food Chem. 65 (10), 2240–2247. 10.1021/acs.jafc.6b05614 28231710

[B51] Zou L. Xu Y. Li Y. He Q. Chen B. Wang D. (2014). Development of a Single-Chain Variable Fragment Antibody-Based Enzyme-Linked Immunosorbent Assay for Determination of Fumonisin B1in Corn Samples. J. Sci. Food Agric. 94 (9), 1865–1871. 10.1002/jsfa.6505 24375282

