# Supplementary material for: Development of Fluorescence Polarization Immunoassay With scFv to Detect Fumonisin Bs in Maize and Simultaneous Study of Their Molecular Recognition Mechanism
Source: Front Chem. 2022 Feb 21;10:829038. doi: 10.3389/fchem.2022.829038 (PMC8900220; doi:10.3389/fchem.2022.829038)
Supplement: Supplementary file 1 [file DataSheet1.pdf]

Supplementary Material

1. Supplementary Figures and Tables

1.1 Supplementary Figures

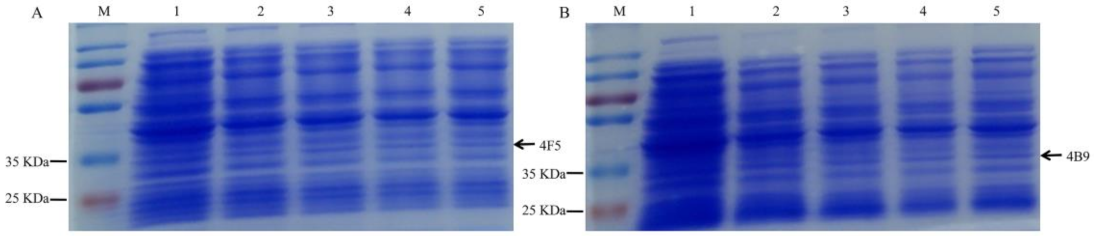

Supplementary Figure 1. Optimization of IPTG concentration for 4F5 scFv and 4B9 scFv expression. (A): The SDS-PAGE of 4F5 scFv, M: marker, 1-5: The concentrations of IPTG were 0, 0.25, 0.5, 0.75, 1.0 mM; (B): the SDS-PAGE of 4B9 scFv, M: marker, 1-5: The concentrations of IPTG were 0, 0.25, 0.5, 0.75, 1.0 mM.

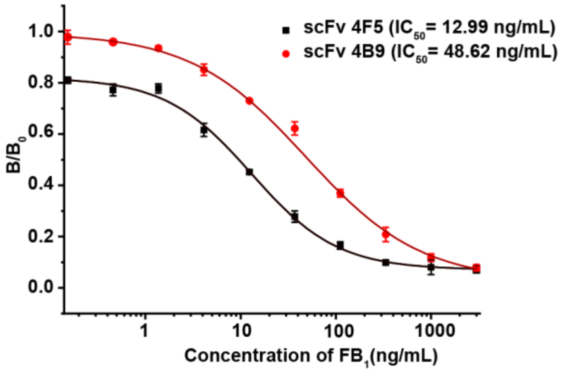

Supplementary Figure 2. Standard curves of the icELISA with 4F5 scFv and 4B9 scFv for the detection of FB_1。

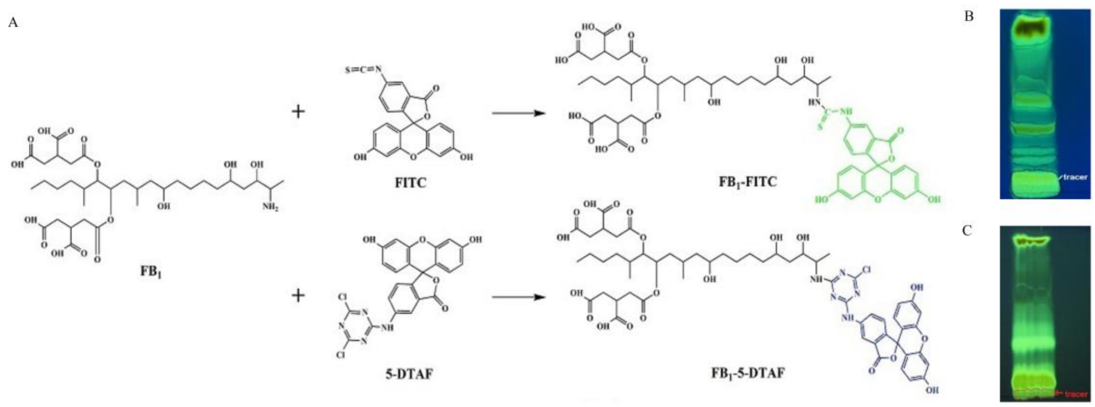

Supplementary Figure 3. The synthesis and purification of FB_1-FITC and FB_1–5-DTAF. (A): The synthesis process of FB_1-FITC and FB_1–5-DTAF; (B) and (C): Purification of FB_1-FITC and FB_1–5-DTAF tracers by thin layer chromatograph.

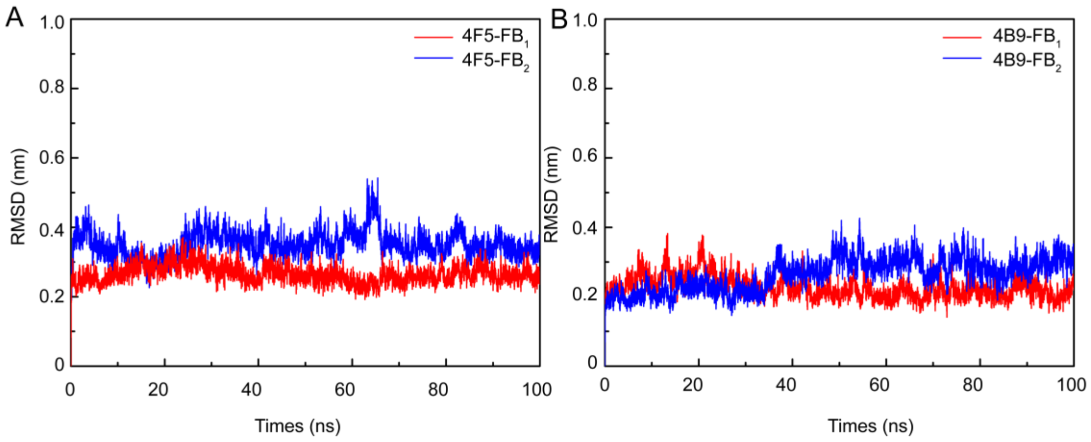

Supplementary Figure 4. The root mean square deviation (RMSD) values of the scFvs-FB$_s$. (A): The RMSD of 4F5-FB$_1$. (B): The RMSD of 4F5-FB$_2$. (C): The RMSD of 4B9-FB$_1$. (D): The RMSD of 4B9-FB$_2$.

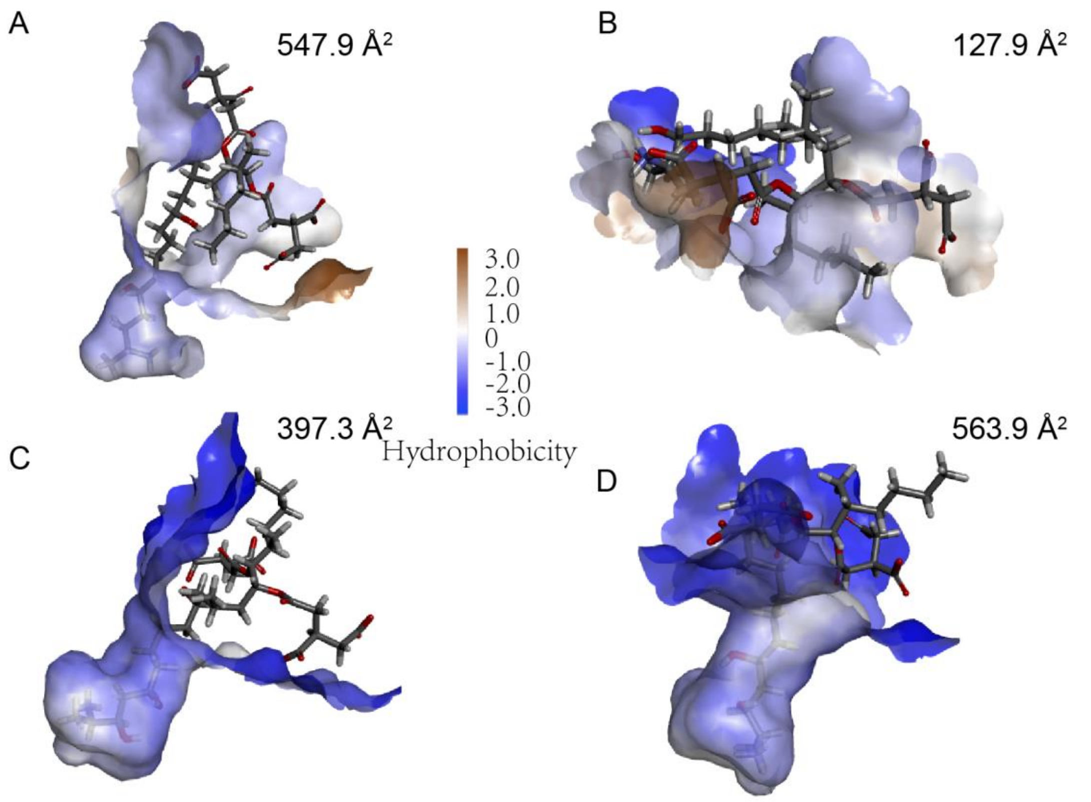

Supplementary Figure 5. The hydrophobicity surface pocket of 4F5-FB$_1$, 4F5-FB$_2$, 4B9-FB$_1$ and 4B9-FB$_2$. (A): The hydrophobicity surface pocket of 4F5-FB$_1$. (B): The hydrophobicity surface pocket of 4F5-FB$_2$. (C): The hydrophobicity surface pocket of 4B95-FB$_1$. (D): The hydrophobicity surface pocket of 4B9-FB$_2$.

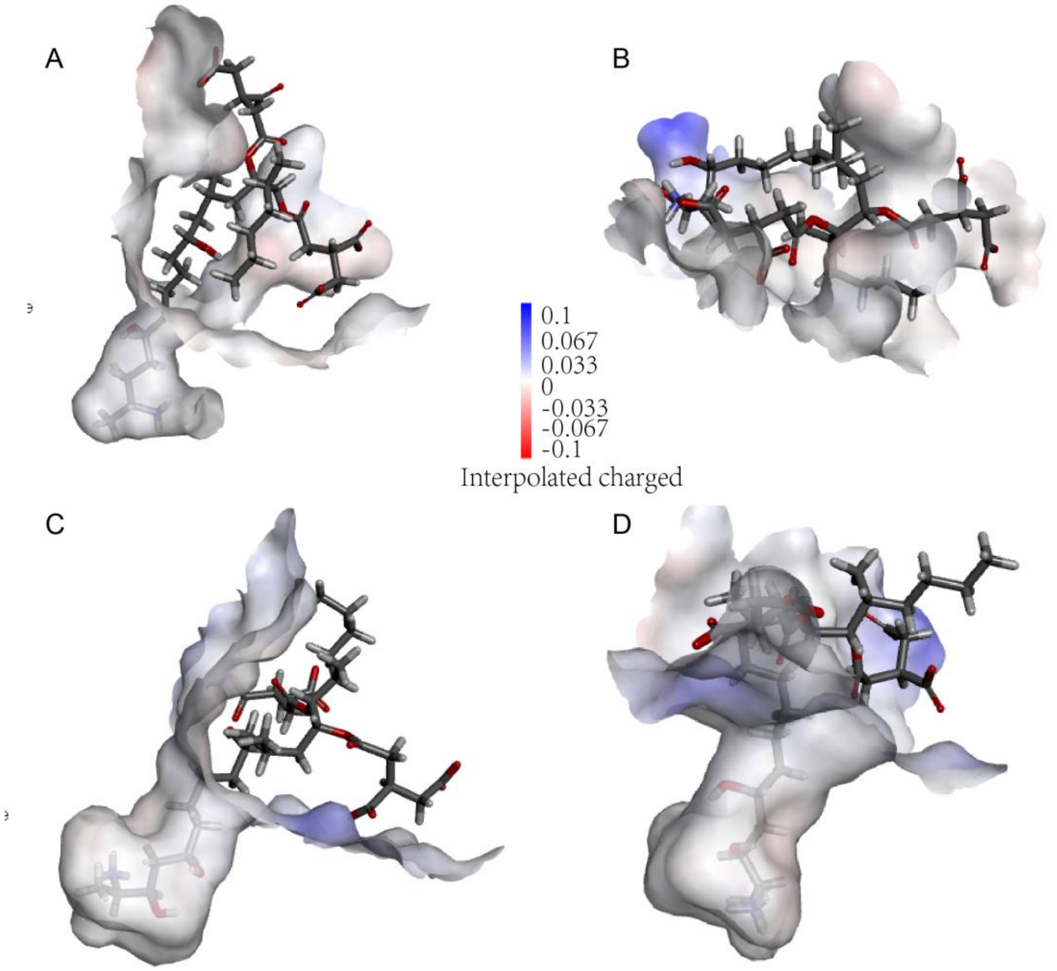

Supplementary Figure 6. The interpolated charged surface pocket of 4F5-FB$_1$, 4F5-FB$_2$, 4B9-FB$_1$ and 4B9-FB$_2$. (A): The interpolated charged surface pocket of 4F5-FB$_1$. (B): The interpolated charged surface pocket of 4F5-FB$_2$. (C): The interpolated charged surface pocket of 4B95-FB$_1$. (D): The interpolated charged surface pocket of 4B9-FB$_2$.

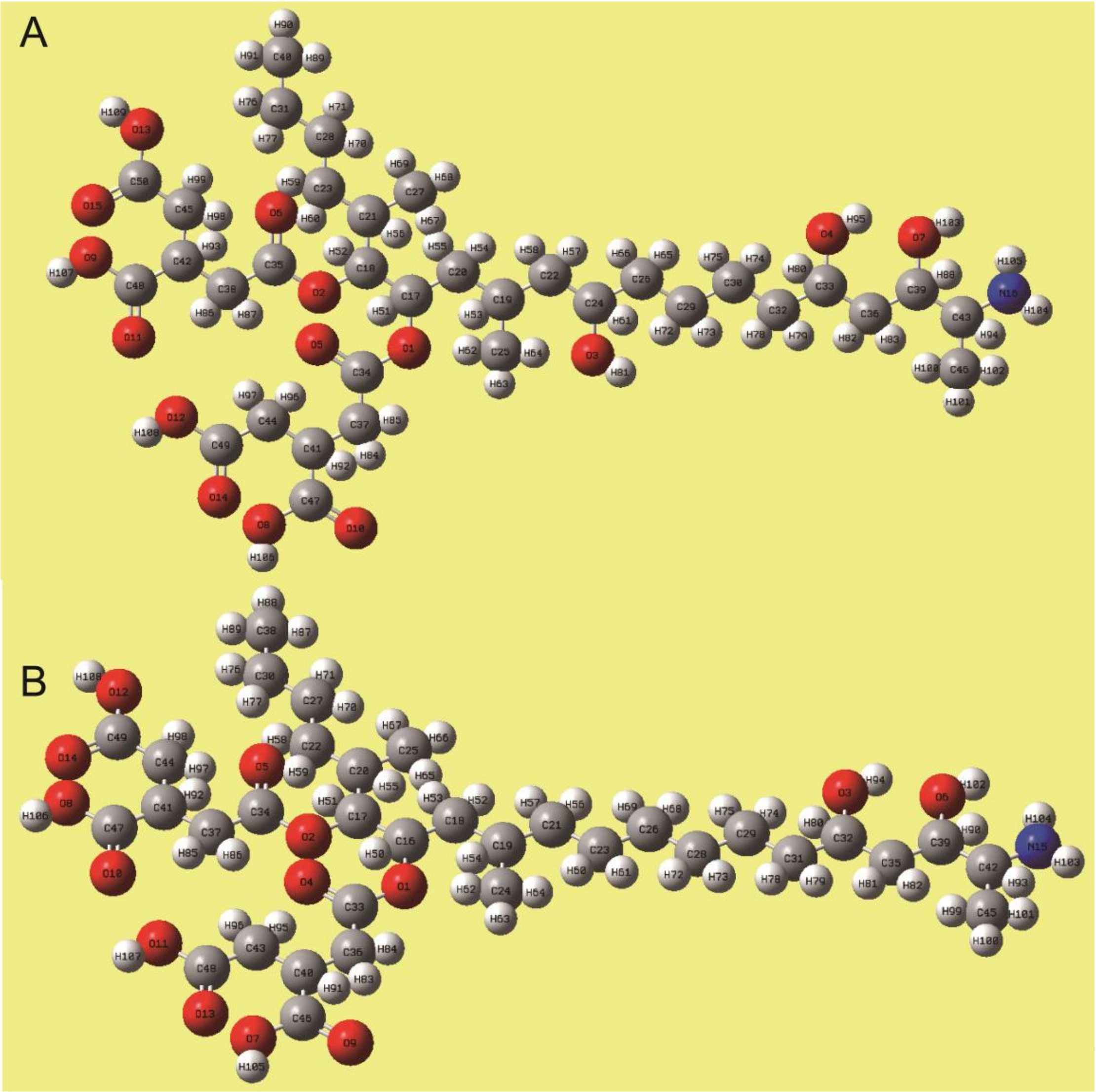

Supplementary Figure 7. The order of the atoms in in the FB_1 and FB_2. (A): The order of the atoms in in the FB_1; (B): The order of the atoms in in the FB_2.

1.2 Supplementary Tables

Table S1. Primers of 4F5 scFv and 4B9 scFv.

Primer	Sequence
4F5-VL-F1	GACATTGTGCTCACCCAATCT
4F5-VL-R1	TTTTATTTCCAGTTTGGTCC
4F5-VL-F2	CTACAGCAGGCCCAGCCGGCCATGGACATTGTGCTCACCCAATCT
4F5-VL-R2	GGAGCCGCCGCCGCCAGAACCACCACCACCAGAACCACCACCACCTTTT ATTTCCAGTTTGGTCC
4F5-VH-F1	GAGGTGAAGCTGGTGGAGTC
4F5-VH-R1	TGAGGAGACGGTGACTGAGG
4F5-VH-F2	GGCGGCGGCGGCTCCGGTGGTGGTGGATCCGAGGTGAAGCTGGTGGAGTC
4B9-VL-F1	GATGTTGTGATGACCCAAACT
4B9-VL-R1	TTTTATTTCCAGCTTGGTCCC
4B9-VL-F2	CTACAGCAGGCCCAGCCGGCCATGGATGTTGTGATGACCCAAACT
4B9-VL-R2	GGAGCCGCCGCCGCCAGAACCACCACCACCAGAACCACCACCACCTTTTA TTTCCAGCTTGGTCCC
4B9-VH-F1	CAGGTCCAACTGCAGCAACC
4B9-VH-R1	TGAGGAGACTGTGAGAGTGG
4B9-VH-F2	GGCGGCGGCGGCTCCGGTGGTGGTGGATCCCAGGTCCAACTGCAGCAACC
4B9-VH-R2	TCGCTAATCAGTTTTTGTTCGGCGGCCGCTGAGGAGACTGTGAGAGTGG
4B9-VH-R3	CGGAGTCAGGCCCCCGAGGCCAGGTCTTCTTCGCTAATCAGTTTT

Table S2. Sequences of 4F5 scFv and 4B9 scFv.

scFv	sequence
4F5	GACATTGTGCTCACCCAATCTCCAGCTTCTTTGGCTGTGTCTCTAGGGCAGAGAGCC<br>ACCTTCTCCTGCAGAGCCAGTGAAAGTGTTGAATATTATGGCACAGGTTTAATGCAG<br>TGGTACCAACAGAAACCAGGACAGCCACCCAAACTCCTCATCTATGCTGCATCCGA<br>CGTAGAATCTGGGGTCCCTGACAGGTTTAGTGGCAGTGGGTCTGGGACAGACTTCA<br>GCCTCAACATCCATCCTGTGGAGGAGGATGATATTGCAATGTATTTCTGTCAGCAAA<br>GTAGGAAGGTGGGGSGGGGSGGGGSTCCGTACACGTTCGGAGGGGGGACCAAACT<br>GGAAATAAAAGAGGTGAAGCTGGTGGAGTCTGGGGGAGGCTTGGTGAAGCCTGGA<br>GGGTCCCTGAAACTCTCCTGTGCAGCCTCTGGATTCACTTTTAGTAGGTTTGCCATG<br>TCTTGGATTCGCCAGACTCCAGAGAAGAGGCTGGAGTGGGTCTCATCCATTATTAGT<br>GTTGGGACCACCTATTATCCAGACAGTGTGAAGGGCCGATTCACCATCTCCAGAGA<br>TAATGACCGGAACATCCTGTACCTGCAAATGAGCAGTCTGAGGTCTGAGGACACGG<br>CCATGTATTACTGTACAAGAGGCCGCGGTACCTACGGCTATGCTATGGACTACTGGG<br>GTCAAGGAACCTCAGTCACCGTCTCCTCA
4B9	GATGTTGTGATGACCCAAACTCCACTCTCCCTGCCTGTCAGTCTTGGAGATCAGGC<br>CTCCATCTCTTGCAGATCTAGTCAGAGCCTTGTACACAGTAATGGAAACACCTATTT<br>ACATTGGTACCTGCAGAAGCCAGGCCAGTCTCCAAAGCTCCTGATCTACAAAGTTT<br>CCAACCGATTTTCTGGGGTCCCAGACAGGTTCAGTGGCAGTGGATCAGGGACAGAT<br>TTCACACTCAAGATCAGCAGAGTGGAGACTGAGGATCTGGGAGTTTATTTCTGCTC<br>TCAAAGTACACATGTTCCGTACACGTTCGGAGGGGGGACCAAGCTGGAAATAAAA<br>GGGGSGGGGSGGGGSCAGGTCCAACTGCAGCAACCTGGGTCTGAGCTGGTGAGGC<br>CTGGAGCTTCAGTGAAGCTGTCCTGCAAGGCCTTTGGCTACAGATTCACCAGCAAC<br>TGGATGCACTGGATGAAGCAGAGGCCTGGACAGGGCCTTGAGTGGATTGGAAAAA<br>TTTATCCTGGGAATTTTACTACTAACTACGATGAGAAGTTCAAGACCAAGGCCACAC<br>TGACTGTAGACACATCCTCCAGCACAGCCTACATGCAGCTCAGCAGCCTGACATCT<br>GACGACTCTGCGGTCTATTACTGTGCAAACTTACGGGGGGACTACTGGGGCCGAGG<br>CACCACTCTCACAGTCTCCTCA

Table S3. Results of tracer binding with scFv (n=3).

	Free tracer			Antibody biding tracer		
	FP (mP)	SD		FP (mP)	SD	△FP (mP)
FB_1-FITC Rf 0.1	50.5	1.4	4F5 scFv	310.9	0.7	260.4
			4B9 scFv	270.26	2.9	219.76
FB_1-5-DTAF Rf 0.1	51.6	1.3	4F5 scFv	131.5	2.3	79.9
			4B9 scFv	142.3	1.8	90.7

Table S4. An overview of immunoassays for FB_s detection.

Strategies	Analytes	Antibody	Time / steps	Homogeneous/ Heterogeneous	LOD in (μg/L, μg/kg)	Sample	Ref
AuNP-LFIA[a]	FB_1	mAb	15 min/one step	Heterogeneous	60	Corn	[1]
AuNP-LFIA	FB_1, FB_2 and FB_3	mAb	15 min/one step	Heterogeneous	11.25 (FB_1)	Maize	[2]
ICS[b]	FB_1	scFv	15 min/one step	Heterogeneous	25 (in buffer)	Maize	[3]
MB-ELISA[c]	FB_1	Mimotope	60 min/two steps	Heterogeneous	11.1	Maize, wheat	[4]
dc-pELISA[d]	FB_1	mAb	120 min/two steps	Heterogeneous	12.5	Corn	[5]
icELISA[e]	FB_1	mAb	70 min/two steps	Heterogeneous	5.4 (in buffer)	Maize	[6]
FPIA[f]	FB_1 and FB_2	mAb	10 s/ one step	Heterogeneous	157.4 (FB_1) and 290.6 (FB_2)	Maize	[7]
FPIA	FB_1 and FB_2	scFv	10 s/one step	Heterogeneous	441.5 (FB_1) and 344.9 (FB_2)	Maize	This study

[a] Colloidal gold nanoparticle based immunochromatographic test strips. [b] Immunochromatographic strip. [c] Microarray-based immunoassay. [d] Direct competitive plasmonic enzyme-linked immunosorbent assay. [e] Indirect competitive enzyme-linked immunosorbent assay. [f] Fluorescence polarization immunoassay.

Table S5. Detection of FB_1+FB_2 using FPIA and HPLC-MS/MS in positive maize samples(N=3).

Samples	FB_s	HPLC-MS/MS ($\mu g\ kg^{-1}$)	FPIA ($\mu g\ kg^{-1}$)	CV (%)
1	FB_1+FB_2	602	662	8.2
2	FB_1+FB_2	67	– [a]	-
3	FB_1+FB_2	– [a]	– [a]	-
4	FB_1+FB_2	– [a]	– [a]	-
5	FB_1+FB_2	328	415	2.4
6	FB_1+FB_2	1082	756	8.8
7	FB_1+FB_2	1668	1350	4.5
8	FB_1+FB_2	525	782	9.1
9	FB_1+FB_2	10422	8964	2.9

Note: [a] Not detected.

Table S6. The amino acid composition of 4F5 scFv.

Amino acid	No.	content	Amino acid	No.	content	Amino acid	No.	content
Ala (A)	12	4.8%	His (H)	1	0.4%	Thr (T)	16	6.4%
Arg (R)	13	5.2%	Ile (I)	10	4.0%	Trp (W)	4	1.6%
Asn (N)	3	1.2%	Leu (L)	16	6.4%	Tyr (Y)	14	5.6%
Asp (D)	11	4.4%	Lys (K)	10	4.0%	Val (V)	15	6.0%
Cys (C)	4	1.6%	Met (M)	6	2.4%	Pyl (O)	0	0.0%
Gln (Q)	11	4.4%	Phe (F)	9	3.6%	Sec (U)	0	0.0%
Glu (E)	11	4.4%	Pro (P)	10	4.0%			
Gly (G)	40	16.0%	Ser (S)	34	13.6%			

Table S7. The amino acid composition of 4B9 scFv.

Amino acid	No.	content	Amino acid	No.	content	Amino acid	No.	content
Ala (A)	7	2.8%	His (H)	4	1.6%	Thr (T)	21	8.5%
Arg (R)	9	3.7%	Ile (I)	6	2.4%	Trp (W)	5	2.0%
Asn (N)	7	2.8%	Leu (L)	20	8.1%	Tyr (Y)	12	4.9%
Asp (D)	10	4.1%	Lys (K)	13	5.3%	Val (V)	15	6.1%
Cys (C)	4	1.6%	Met (M)	4	1.6%	Pyl (O)	0	0.0%
Gln (Q)	13	5.3%	Phe (F)	9	3.7%	Sec (U)	0	0.0%
Glu (E)	6	2.4%	Pro (P)	10	4.1%			
Gly (G)	37	15.0%	Ser (S)	34	13.8%			

Table S8. Detailed information about the predicted interactions between scFvs and FB_s.

Complex	Category	Types	From	To	Distance (Å)
	Hydrogen Bond	Conventional Hydrogen Bond [a]	H: ARG108	FB_1: O15	1.77
	Hydrogen Bond	Conventional Hydrogen Bond	H: ARG108	FB_1: O13	1.85
	Hydrogen Bond	Conventional Hydrogen Bond	H: THR110	FB_1:O10	1.63
	Hydrogen Bond	Conventional Hydrogen Bond	H: TYR111	FB_1:H101	1.89
	Hydrogen Bond	Conventional Hydrogen Bond	H: TYR113	FB_1:O3	2.04
4F5-FB_1	Hydrogen Bond	Conventional Hydrogen Bond	H: TYR113	FB_1:H103	1.70
	Hydrogen Bond	Conventional Hydrogen Bond	H: MET115	FB_1:O7	2.51
	Hydrogen Bond	Conventional Hydrogen Bond	L: TYR55	FB_1:O11	1.63
	Hydrogen Bond	Conventional Hydrogen Bond	L: GLN105	FB_1:O4	1.99
	Hydrogen Bond	Conventional Hydrogen Bond	L: GLN105	FB_1:H106	1.96
	Hydrogen Bond	Conventional Hydrogen Bond	L: TYR42	FB_1:H104	2.00
	Hydrogen Bond	Conventional Hydrogen Bond	L: ARG108	FB_2:O10	1.78
	Hydrogen Bond	Conventional Hydrogen Bond	L: TYR31	FB_2:O13	1.67
	Hydrogen Bond	Conventional Hydrogen Bond	L: TYR34	FB_2:O13	1.61
4F5-FB_2	Electrostatic force	Attractive Charge [b]	L: LYS109	FB_2:O14	2.79
	Electrostatic force	Attractive Charge	L: ASP1	FB_2:O14	5.08
	Hydrophobic force	Pi-Alkyl [c]	L: TYR31	FB_2:C25	4.34
	Hydrogen Bond	Conventional Hydrogen Bond	H: TRP38	FB_1:O12	1.72
	Hydrogen Bond	Conventional Hydrogen Bond	L: HIS31	FB_1:O5	2.43
	Hydrogen Bond	Conventional Hydrogen Bond	L: TYR38	FB_1:O6	1.70
	Hydrogen Bond	Conventional Hydrogen Bond	L: TYR55	FB_1:O9	1.74
	Hydrogen Bond	Conventional Hydrogen Bond	L: TYR55	FB_1:O13	2.74
	Hydrogen Bond	Conventional Hydrogen Bond	L: SER105	FB_1:H104	1.81
	Hydrogen Bond	Conventional Hydrogen Bond	L: SER106	FB_1:H105	1.76
	Hydrogen Bond	Conventional Hydrogen Bond	L: TYR116	FB_1:H106	1.88
	Electrostatic force	Salt bridge [d]	H: LYS55	FB_1: O8	2.36
4B9-FB_1	Electrostatic force	Salt bridge	H: LYS55	FB_1: O14	1.81
	Electrostatic force	Salt bridge	L: LYS56	FB_1:O9	1.93
	Electrostatic force	Salt bridge	L: LYS56	FB_1:O11	2.09
	Electrostatic force	Attractive charge	L: ARG108	FB_1:O13	4.50
	Electrostatic force	Pi-cation [e]	L: PHE118	FB_1:N16	4.29
	Hydrophobic force	Pi-Alkyl	L: HIS31	FB_1:C27	4.94
	Hydrophobic force	Pi-Alkyl	L: HIS31	FB_1:C25	4.44
	Hydrophobic force	Pi-Alkyl	L: TYR38	FB_1:C25	4.28
	Hydrogen Bond	Conventional Hydrogen Bond	H: ARG108	FB_2:H103	2.10
	Hydrogen Bond	Conventional Hydrogen Bond	H: ARG108	FB_2:H104	1.96
	Hydrogen Bond	Conventional Hydrogen Bond	L: TYR55	FB_2:H101	3.01
	Hydrogen Bond	Conventional Hydrogen Bond	L: TYR55	FB_2: O10	2.72
4B9-FB_2	Hydrogen Bond	Conventional Hydrogen Bond	L: TYR55	FB_2: O12	1.72
	Hydrogen Bond	Conventional Hydrogen Bond	L: TYR56	FB_2: O12	1.81
	Hydrogen Bond	Conventional Hydrogen Bond	L: TYR38	FB_2: O14	1.69
	Hydrogen Bond	Conventional Hydrogen Bond	H: TYR38	FB_2:O11	1.80

Hydrogen Bond	Conventional Hydrogen Bond	H: LYS55	FB_2: O7	2.26
Electrostatic	Salt bridge	L: LYS56	FB_2: O14	2.86
Electrostatic	Salt bridge	H: LYS55	FB_2: O9	1.88
Electrostatic	Salt bridge	H: LYS55	FB_2: O13	1.74
Electrostatic	Pi-Alkyl	L: HIS31	FB_2: N15	4.64

[a] is a bond that Protein-donating OH or NH group approaches the lone pair of an O or N atom. [b] is between two oppositely charged objects. [c] is interaction of pi- electron cloud over an aromatic group and electron group of any alkyl group. [d] is a combination of two non-covalent interactions: hydrogen bonding and ionic bonding. [e] is the interaction between an electron deficient (π-acidic) aromatic system and an anion.

1. Hou, S.; Ma, J.; Cheng, Y.; Wang, H.; Yan, Y., One-stop rapid detection of fumonisin B1, dexyonivalenol and zearalenone in grains. *Food Control* **2020,** *117*, 107107.

2. Yao, J.; Sun, Y.; Li, Q.; Wang, F.; Teng, M.; Yang, Y.; Deng, R.; Hu, X., Colloidal gold‐McAb probe‐based rapid immunoassay strip for simultaneous detection of fumonisins in maize. *Journal of the Science of Food & Agriculture* **2016**.

3. Ren, W.; Xu, Y.; Huang, Z.; Li, Y.; Tu, Z.; Zou, L.; He, Q.; Fu, J.; Liu, S.; Hammock, B. D., Single-chain variable fragment antibody-based immunochromatographic strip for rapid detection of fumonisin B1 in maize samples. *Food chemistry* **2020,** *319*, 126546.

4. Peltomaa, R.; Benito-Peña, E.; Barderas, R.; Sauer, U.; Andrade, M. G.; Moreno-Bondi, M. C., Microarray-Based Immunoassay with Synthetic Mimotopes for the Detection of Fumonisin B1. *Anal. Chem.* **2017**.

5. Chen; Xirui; Liang; Yi; Zhang; Wenjing; Leng; Yuankui; Xiong; Yonghua, A colorimetric immunoassay based on glucose oxidase-induced AuNP aggregation for the detection of fumonisin B-1. *Talanta the International Journal of Pure & Applied Analytical Chemistry* **2018**.

6. Sheng, Y.; Jiang, W.; De Saeger, S.; Shen, J.; Zhang, S.; Wang, Z., Development of a sensitive enzyme-linked immunosorbent assay for the detection of fumonisin B1 in maize. *Toxicon* **2012,** *60* (7), 1245-1250.

7. Li, C.; Mi, T.; Conti, G. O.; Yu, Q.; Wang, Z., Development of a Screening Fluorescence Polarization Immunoassay for the Simultaneous Detection of Fumonisins B1 and B2 in Maize. *Journal of Agricultural & Food Chemistry* **2015,** *63* (20), 4940.
